# Free Fatty Acid Receptor 4 (FFA4) Activation Ameliorates Imiquimod-Induced Psoriasis in Mice

**DOI:** 10.3390/ijms23094482

**Published:** 2022-04-19

**Authors:** So-Eun Son, Jung-Min Koh, Dong-Soon Im

**Affiliations:** 1 Department of Biomedical and Pharmaceutical Sciences, Graduate School, Kyung Hee University, Seoul 02447, Korea; seson@khu.ac.kr; 2 Division of Endocrinology and Metabolism, Asan Medical Center, University of Ulsan College of Medicine, Seoul 05505, Korea; jmkoh@amc.seoul.kr; 3 Department of Basic Pharmaceutical Sciences, Graduate School, Kyung Hee University, Seoul 02447, Korea

**Keywords:** psoriasis, imiquimod, free fatty acid receptor 4, FFA4, polyunsaturated fatty acids, IL-17, skin, GPR120

## Abstract

Dietary supplementation with n-3 polyunsaturated fatty acids (n-3 PUFA) has been used as an adjunct therapy for psoriasis due to its anti-inflammatory properties. Free fatty acid receptor 4 (FFA4 or GPR120) is a receptor-sensing n-3 PUFA. In the present study, we examined whether FFA4 acted as a therapeutic target for n-3 PUFA in psoriasis therapy. Experimentally, psoriasis-like skin lesions were induced by treatment with imiquimod for 6 consecutive days. A selective FFA4 agonist, Compound A (30 mg/kg), was used in FFA4 WT and FFA4 KO mice. Imiquimod-induced psoriasis-like skin lesions, which present as erythematous papules and plaques with silver scaling, as well as markedly elevated IL-17/IL-23 cytokine levels in skin tissues, were significantly suppressed by Compound A in FFA4 WT mice, but not in FFA4 KO mice. Enlarged lymph nodes and spleens, as well as imiquimod-induced, elevated IL-17/IL-23 cytokine levels, were also strongly suppressed by Compound A in FFA4 WT mice, but not in FFA4 KO mice. Imiquimod-induced increases in the CD4^+^IL-17A^+^ T cell population in lymph nodes and spleens were suppressed by Compound A treatment in FFA4 WT mice; however, this was not seen in FFA4 KO mice. Furthermore, compound A suppressed the differentiation of CD4^+^ naïve T cells from splenocytes into T_H_17 cells in an FFA4-dependent manner. In conclusion, we demonstrated that the activation of FFA4 ameliorates imiquimod-induced psoriasis, and the suppression of the differentiation of T_H_17 cells may partly contribute to its efficacy. Therefore, we suggest that FFA4 could be a therapeutic target for psoriasis therapy.

## 1. Introduction

Psoriasis is a complex auto-immune skin disorder typically characterized by erythematous papules and plaques with silver scaling [1]. In psoriasis, characteristic dermal inflammation and epidermal hyperplasia with abnormal keratinocyte hyperproliferation are triggered and maintained by keratinocytes and immune cells [2,3]. The initial discovery of T_H_1 cells and T_H_1 cytokines and later findings of T_H_17 cells and T_H_17 cytokines in psoriatic skin lesions support the pathogenesis of psoriasis [4,5,6,7,8]. Additionally, T_H_17 and T_H_1 cells are believed to play primary pathogenic roles in human psoriasis [9]. Previous and current data show that T_H_17 and T_H_1 cells are present within the psoriatic skin tissues [9]. Moreover, pro-inflammatory metabolites from arachidonic acid, such as leukotriene B_4_ and 12-hydroxyeicosatetraenoic acid, are high in psoriatic plaques [10]. Thus, dietary fish oils containing n-3 polyunsaturated fatty acids (n-3 PUFA) have been used as adjunct therapies, owing to their anti-inflammatory properties [11,12].

On the other hand, a systematic review found that the use of n-3 PUFA in patients with psoriasis is associated with psoriasis amelioration and other outcomes; there are both positive and negative results from many randomized controlled trials [13]. Some studies found improvements in the following psoriatic parameters after n-3 PUFA intake: Psoriasis area severity index (PASI) score, erythema, scaling, itching, area involved and infiltration; in contrast, some studies found no significant effect on the same parameters [13]. Different study durations and treatment doses could be possible causes for this discrepancy (e.g., durations from 10 days to 1 year and doses ranging from 0.216 to 5.400 g of eicosapentaenoic acid (EPA) and 0.132 to 4.200 g of docosahexaenoic acid (DHA)) [11,12,14,15,16]. 

Recently, a novel molecular mode of action has been added in n-3 PUFA pharmacology: free fatty acid receptor 4 (FFA4 or GPR120) [17,18]. FFA4 is a G protein-coupled receptor located on the cell membrane and it works through activation of G_q/11_ and G_12/13_ proteins [17,19]. Its phosphorylation happens when it activates GRK and PKC [20]. Its natural ligands are long-chain polyunsaturated fatty acids, such as DHA and EPA [17]. The expression of FFA4 has been reported in immune cells, including monocytes, macrophages, dendritic cells and eosinophils [21,22]. Furthermore, FFA4 was expressed in intact skin, particularly in keratinocytes; however, no expression in fibroblasts was noted [23]. DHA decreased the expression of IL-1β, but increased that of IL-6, TGF-β and the keratinocyte marker, involucrin [23]. Furthermore, high TLR expression has been detected in psoriatic tissues and peripheral blood mononuclear cells of patients with psoriasis [24]. Experimentally, the application of topical imiquimod, a TLR7/8 ligand, exhibits pro-psoriatic actions via the IL-23/IL-17 axis [24,25,26]. In this study, we examined whether FFA4 is a molecular target of n-3 PUFA treatment for psoriasis by using an imiquimod-induced psoriasis model in combination with Compound A (3-[2-chloro-5-(trifluoromethoxy)phenyl]-3-azaspiro[5.5]undecane-9-acetic acid), a selective FFA4 agonist, in FFA4 WT and FFA4 KO mice [27,28].

## 2. Results

### 2.1. Compound A Suppressed Imiquimod-Induced Psoriasis in FFA4 WT Mice, but Not in KO Mice

**Figure 1:**
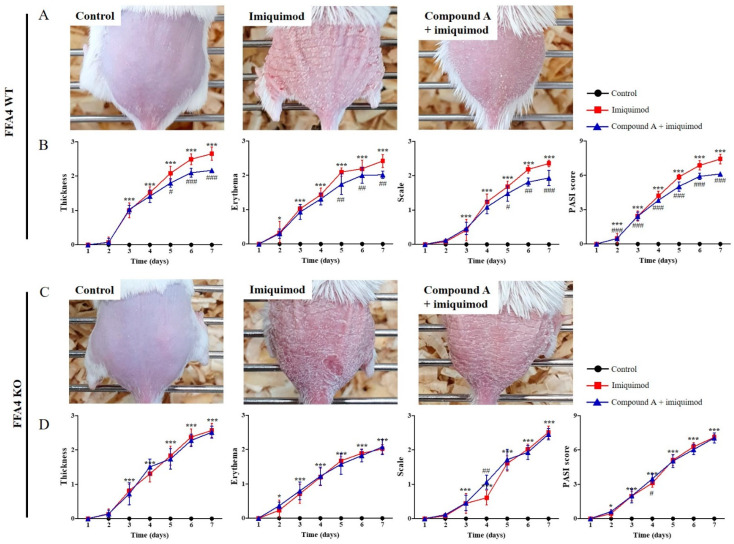
Administration of Compound A suppressed imiquimod-induced psoriasis-like skin inflammation in FFA4 WT mice, but not in FFA4 KO mice. A murine model of imiquimod-induced psoriasis was established through consecutive imiquimod application for 6 days; Compound A (30 mg/kg, i.p.) was administrated 30 min before imiquimod application to FFA4 WT BALB/c mice (**A**,**B**) or FFA4 KO mice (**C**,**D**). (**A**,**C**) Macroscopic views of mouse back skin after 6 days of treatment. (**B**,**D**) Thickening, erythema, scale and PASI score, which is the sum of thickening, erythema and scaling scores on the seventh day. Two-way analysis of variance with Bonferroni multiple comparison test was conducted. The results are presented as the means ± SEM (n = 5). *** *p* < 0.001, * *p* < 0.05 vs. the vehicle-treated group, ^###^ *p* < 0.001, ^##^ *p* < 0.01, ^#^ *p* < 0.05 vs. the imiquimod-treated group.

N-3 PUFA could exert their effects via competition on COX and LOX with arachidonic acid, but Compound A can’t compete. Also, n-3 PUFA could be converted into pro-resolving mediators, such as resolvin Es and Ds, but Compound A can’t be converted. Thus, instead of n-3 PUFA, we treated mice with Compound A using an in vivo, imiquimod-induced psoriasis model in FFA4 WT BALB/c mice. After 6 days of treatment, imiquimod-induced psoriatic skin lesions were clearly observed; Compound A administration suppressed the erythematous papules and plaques with silver scaling (Figure 1A). Three days after the start of imiquimod application on the shaved back skin, signs of thickening, erythema and scaling appeared, which continually increased in severity until the end of the experiment (Figure 1B). However, the administration of Compound A significantly suppressed the signs and the PASI scores (Figure 1B).

To verify the involvement of FFA4 in the efficacy of Compound A, the same experiment was conducted in FFA4 KO BALB/c mice (Figure 1C). After 6 days of treatment, imiquimod-induced psoriatic skin lesions were clearly observed; however, Compound A administration did not suppress the erythematous papules and plaques with silver scaling in FFA4 KO mice (Figure 1C). Three days after the start of imiquimod application on the shaved back skin, signs of thickening, erythema and scaling appeared, which continued to increase in severity until the end of the experiment; however, administration of Compound A did not suppress the signs and the PASI scores in FFA4 KO mice (Figure 1D).

### 2.2. Compound A Suppressed Imiquimod-Induced Keratinocyte Proliferation in FFA4 WT Mice, but Not in KO Mice

**Figure 2:**
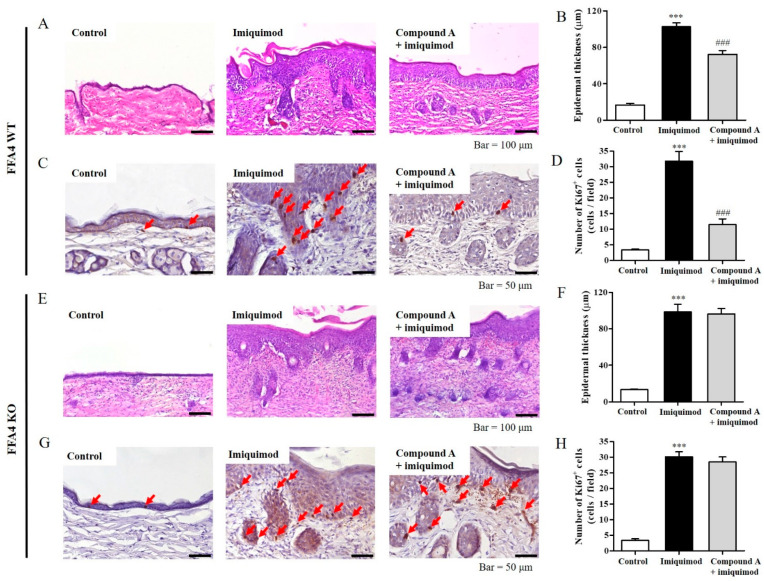
Administration of compound A suppressed imiquimod-induced pathologic skin changes in FFA4 WT mice, but not in FFA4 KO mice. Imiquimod was applied on the back skin for 6 consecutive days; Compound A (30 mg/kg, i.p.) was administrated 30 min before imiquimod application to FFA4 WT mice (**A**–**D**) or FFA4 KO mice (**E**–**H**). (**A**,**E**) H&E staining of the back skin. In the imiquimod group, we detected lengthening and clubbing of the rete ridges and moderate-to-severe dermal lymphocyte infiltrates. (**B**,**F**) Epidermal thickness. (**C**,**G**) Ki-67 staining was evaluated from 5 sample images per mouse. Ki-67-positive cells are indicated by red arrows. (**D**,**H**) Histograms of Ki-67^+^ cell numbers. The results are presented as the means ± SEM (n = 5). *** *p* < 0.001 vs. the vehicle-treated group, ^###^ *p* < 0.001 vs. the imiquimod-treated group.

Subsequently, skin samples were stained with H&E and Ki-67. H&E staining revealed thickening of the epidermis due to hyperkeratosis in imiquimod-treated mice compared to the control group mice (Figure 2A). Extensive imiquimod-induced hypertrophy of the epidermis of the skin was significantly suppressed by Compound A treatment in the FFA4 WT mice, as shown by histograms of epidermal tissue thickness (Figure 2B). A major symptom of psoriasis is keratinocyte hyperproliferation. Compared with the control group, the imiquimod group showed a significant increase in Ki-67-positive keratinocytes (Figure 2C). However, sections from mice in the imiquimod plus Compound A-treated group had fewer Ki-67-positive cells and less epidermal thickness (Figure 2C,D). On the other hand, in FFA4 KO mice, H&E and Ki-67 staining showed imiquimod-induced thickening of the epidermis and hyperproliferation of keratinocytes (Figure 2E–H). However, in this group, the imiquimod-induced increase in Ki-67-positive keratinocytes and epidermal thickness was no longer suppressed by Compound A (Figure 2E–H).

### 2.3. Compound A Suppressed Imiquimod-Induced Production of T_H_17 Cytokines in the Skin of FFA4 WT Mice, but Not in the Skin of KO Mice

**Figure 3:**
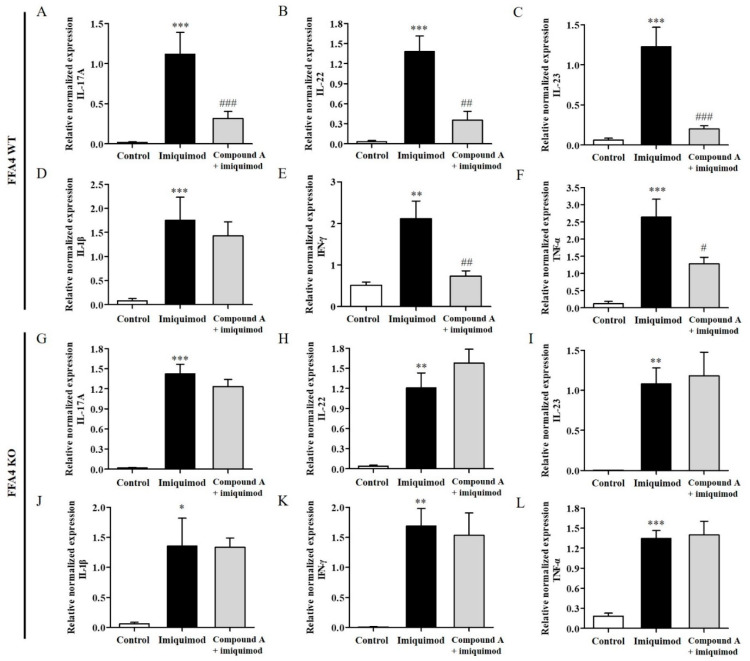
Effect of Compound A on the expression levels of the T_H_17 and T_H_1 cytokines in the skin tissues of imiquimod-induced psoriasis in FFA4 WT and KO mice. qRT-PCR analysis of the mRNA expression of T_H_17 (IL-17A, IL-22 and IL-23) and T_H_1 (IL-1β, IFN-γ and TNF-α) cytokines in skin tissues from the imiquimod-induced and Compound A-treated FFA4 WT (**A**–**F**) or FFA4 KO (**G**–**L**) groups. (**A**,**G**) IL-17A, (**B**,**H**) IL-22, (**C**,**I**) IL-23, (**D**,**J**) IL-1β, (**E**,**K**) IFN-γ and (**F**,**L**) TNF-α. The relative mRNA levels of the cytokines were quantified by determining the ratios of their levels to GAPDH transcript levels. The results are presented as the mean ± SEM (n = 5). *** *p* < 0.001, ** *p* < 0.01, * *p* < 0.05 vs. the vehicle-treated group, ^###^ *p* < 0.001, ^##^ *p* < 0.01, ^#^ *p* < 0.05 vs. the imiquimod-treated group.

The levels of inflammatory T_H_17 cytokines (IL-17A, IL-22 and IL-23) and T_H_1 cytokines (IL-1β, IFN-γ and TNF-α) were also measured in the skin; psoriasis is considered to be regulated by T_H_17 and T_H_1 responses. The mRNA levels of all cytokines were significantly elevated in the skin tissues of FFA4 WT mice (Figure 3A–F). The increase in the levels of inflammatory T_H_17 cytokines (IL-17A, IL-22 and IL-23) in the skin was significantly suppressed by Compound A treatment in FFA4 WT mice (Figure 3A–C). Furthermore, the increase in the levels of inflammatory T_H_1 cytokines (IFN-γ and TNF-α) in the skin was significantly suppressed by Compound A treatment in FFA4 WT mice, but the increase in IL-1β mRNA levels was not suppressed (Figure 3D–F). In FFA4 KO mice, the mRNA levels of T_H_17 and T_H_1 cytokines also increased in the skin after imiquimod treatment; however, Compound A treatment proved ineffective in this group (Figure 3G–L).

### 2.4. Compound A Suppressed Imiquimod-Induced Psoriasis Responses in the Lymph Nodes of FFA4 WT Mice, but Not in the Lymph Nodes of KO Mice

**Figure 4:**
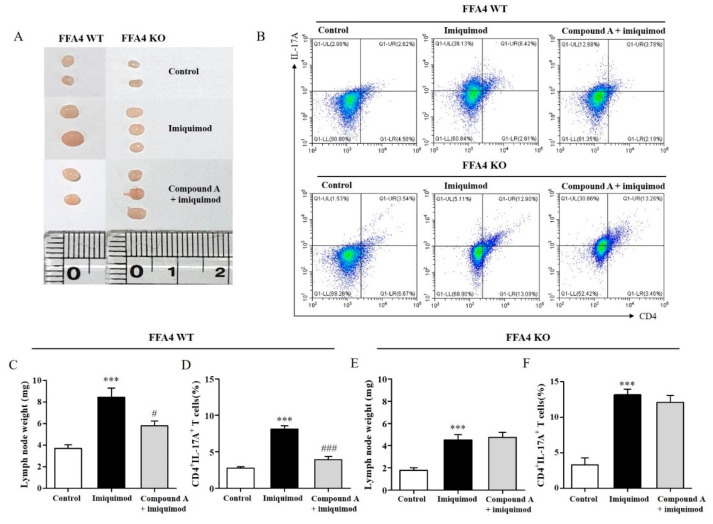
Effect of Compound A on the lymph node sizes and CD4^+^IL-17A^+^ T cell population in FFA4 WT and KO mice. (**A**) The draining lymph nodes were photographed to record morphological changes. (**B**) Representative flow cytometry dot plots of the FACS analysis of CD4^+^IL-17A^+^ T cells. (**C**,**E**) The measured weights of lymph nodes. (**D**,**F**) Percentage of CD4^+^IL-17A^+^ T cells in the lymph nodes. Data were tested for Gaussian distribution using the KS normality test. The results are presented as the mean ± SEM (n = 5). *** *p* < 0.001 vs. the vehicle-treated group, ^###^ *p* < 0.001, ^#^ *p* < 0.05 vs. the imiquimod-treated group.

Next, we checked the sizes of the lymph nodes as well as the CD4^+^IL-17A^+^ T cell population in these organs. Draining lymph nodes orchestrates an immune response in the skin. Lymph nodes become swollen when an infection or an immune reaction occurs. The lymph nodes in the imiquimod-induced psoriasis mouse group were much more swollen than those in the control group, as manifested by increased lymph node weights (Figure 4A,C). Compound A significantly suppressed the imiquimod-induced increase in lymph node weight by 55.9% in the WT mice (Figure 4A,C). The number of CD4^+^IL-17A^+^ T cells in the lymph nodes was also measured. According to FACS analysis of isolated cells from the draining lymph nodes, the CD4^+^IL-17A^+^ T cell numbers were increased by imiquimod but suppressed by Compound A (Figure 4B,D). In imiquimod-treated FFA4 KO mice, the lymph nodes became swollen and were accompanied by increased lymph node weights (Figure 4A,E). In this group, Compound A did not suppress the imiquimod-induced increase in lymph node weight (Figure 4A,E). Furthermore, the number of CD4^+^IL-17A^+^ T cells in the lymph nodes was increased by imiquimod but was not suppressed by Compound A (Figure 4B,F). Lymph node weights of the control group in FFA4 KO mice were smaller than those in FFA4 WT mice (WT 3.7 mg vs. KO 1.8 mg), but imiquimod-induced increase of lymph node weights in FFA4 KO mice was higher than those in FFA4 WT mice (WT 128% vs. KO 154%). There was no significant difference in the percentages of CD4^+^IL17^+^ T cells in the control groups (WT 2.8% vs. KO 3.3%), but the imiquimod-induced increase of CD4^+^IL17^+^ T cells was much higher in FFA4 KO mice than in FFA4 WT mice (WT 193% vs. KO 298%).

### 2.5. Compound A Suppressed Imiquimod-Induced Psoriasis Production of T_H_17 Cytokines in the Lymph Nodes of FFA4 WT Mice, but Not in the Lymph Nodes of KO Mice

**Figure 5:**
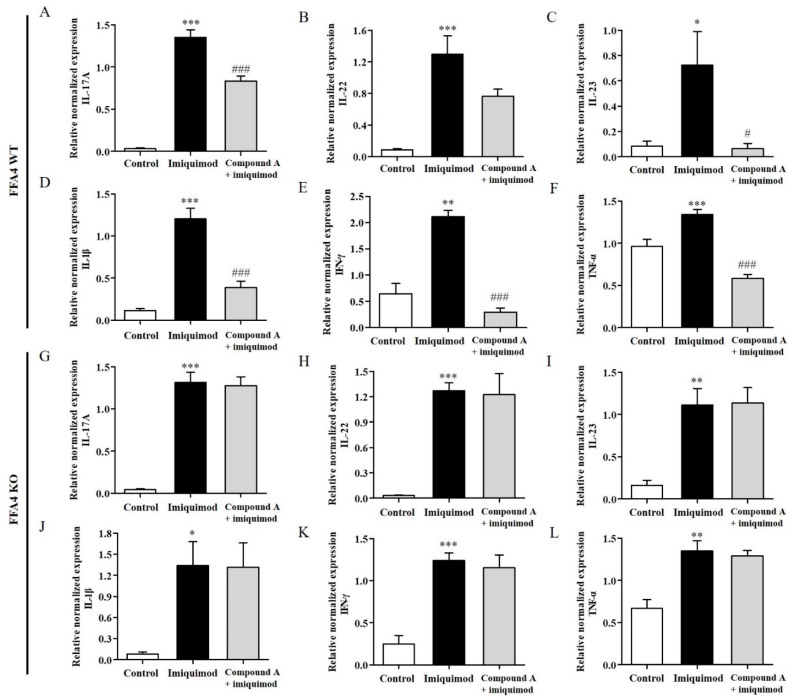
Effect of Compound A on the expression levels of the T_H_17 and T_H_1 cytokines in the lymph nodes of imiquimod-induced psoriasis in FFA4 WT and KO mice. qRT-PCR analysis of the mRNA expression of T_H_23/T_H_17 axis (IL-17A, IL-22 and IL-23) and T_H_1 (IL-1β, IFN-γ and TNF-α) cytokines in lymph nodes from the imiquimod-induced and Compound A-treated FFA4 KO (**A**–**F**) or FFA4 KO (**G**–**L**) groups. (**A**,**G**) IL-17A, (**B**,**H**) IL-22, (**C**,**I**) IL-23, (**D**,**J**) IL-1β, (**E**,**K**) IFN-γ and (**F**,**L**) TNF-α. The relative mRNA levels of the cytokines were quantified by determining the ratios of their levels to GAPDH transcript levels. The results are presented as the mean ± SEM (n = 5). *** *p* < 0.001, ** *p* < 0.01, * *p* < 0.05 vs. the vehicle-treated group, ^###^ *p* < 0.001, ^#^ *p* < 0.05 vs. the imiquimod-treated group.

The levels of inflammatory T_H_23/T_H_17 axis cytokines (IL-17A, IL-22 and IL-23) and T_H_1 cytokines (IL-1β, IFN-γ and TNF-α) were measured in the lymph nodes. The mRNA levels of these cytokines were significantly elevated in the lymph nodes after psoriasis induction in WT mice; Compound A treatment suppressed the elevation of cytokine levels (Figure 5A–F), although significance was not observed for IL-22 levels (Figure 5B). In FFA4 KO mice, the levels of inflammatory T_H_23/T_H_17 axis cytokines (IL-17A, IL-22 and IL-23) and T_H_1 cytokines (IL-1β, IFN-γ and TNF-α) in the lymph nodes were also increased after psoriasis induction; however, Compound A treatment did not suppress the cytokine levels (Figure 5G–L).

### 2.6. Compound A Suppressed Imiquimod-Induced Psoriasis Responses in the Spleens of FFA4 WT Mice, but Not in the Spleens of KO Mice

**Figure 6:**
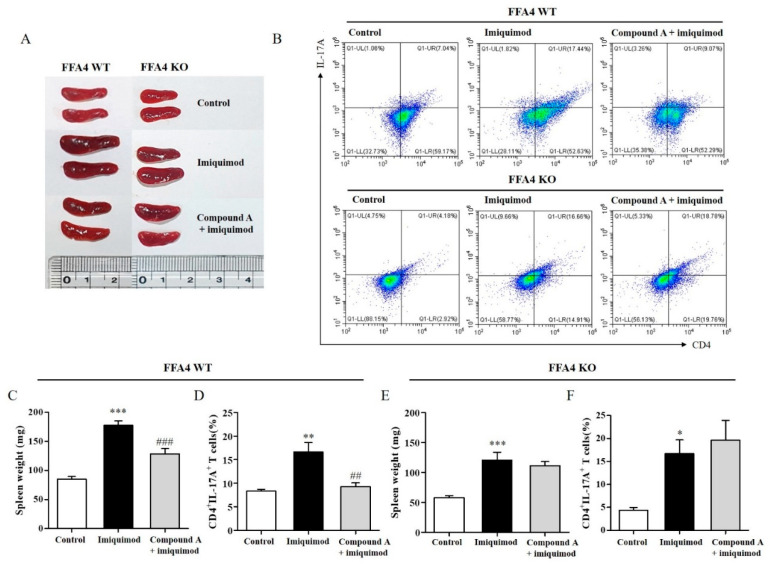
Effect of Compound A on the spleen sizes and CD4^+^IL-17A^+^ T cell population in FFA4 WT and KO mice. (**A**) The spleens were photographed to record morphological changes. (**B**) Representative flow cytometry dot plots of the FACS analysis of CD4^+^IL-17A^+^ T cells. (**C**,**E**) The measured weights of spleens. (**D**,**F**) Percentage of CD4^+^IL-17A^+^ T cells in the spleens. Data was tested for Gaussian distribution using the KS normality test. The results are presented as the mean ± SEM (n = 5). *** *p* < 0.001, ** *p* < 0.01, * *p* < 0.05 vs. the vehicle-treated group, ^###^ *p* < 0.001, ^##^ *p* < 0.001 vs. the imiquimod-treated group.

The size of the spleen and the CD4^+^IL-17^+^ T cell population were also measured. The spleen is analogous to the large lymph nodes and plays an important role in the immune response. Splenomegaly can result from infections or immune reactions. In the imiquimod-induced psoriasis mouse group, the spleens became enlarged and increased in weight compared to those in the control group (Figure 6A,C). Compound A significantly suppressed the imiquimod-induced increase in spleen weight by 53% in WT mice (Figure 6A,C). The number of CD4^+^IL-17A^+^ T cells in the spleen was measured. According to FACS analysis of isolated cells from the spleen, the CD4^+^IL-17A^+^ T cell numbers were increased by imiquimod and suppressed by Compound A (Figure 6B,D). In FFA4 KO mice, the size of the spleen and the CD4^+^IL-17A^+^ T cell population were also increased by imiquimod (Figure 6A,E); however, Compound A did not suppress the imiquimod-induced increase in spleen weight (Figure 6A,E). The numbers of CD4^+^IL-17A^+^ T cells in the spleen were also increased by imiquimod; however, Compound A did not suppress the increased CD4^+^IL-17A^+^ T cell population (Figure 6B,F). Spleen weights of the control group in FFA4 KO mice were smaller than those in FFA4 WT mice (WT 85 mg vs. KO 58 mg), but the imiquimod-induced increase rate in spleen weights was the same in FFA4 KO mice as it was in FFA4 WT mice (WT 109% vs. KO 109%). Percentages of CD4^+^IL17^+^ T cells in the control groups in FFA4 KO mice were smaller than those in FFA4 WT mice (WT 8.4% vs. KO 4.4%), but the imiquimod-induced increase rate of CD4^+^IL17^+^ T cells was much higher in FFA4 KO mice than in FFA4 WT mice (WT 99% vs. KO 291%).

### 2.7. Compound A Suppressed Differentiation into T_H_17 Cells from Naïve T Cells of FFA4 WT Mice, but Not KO Mice

**Figure 7:**
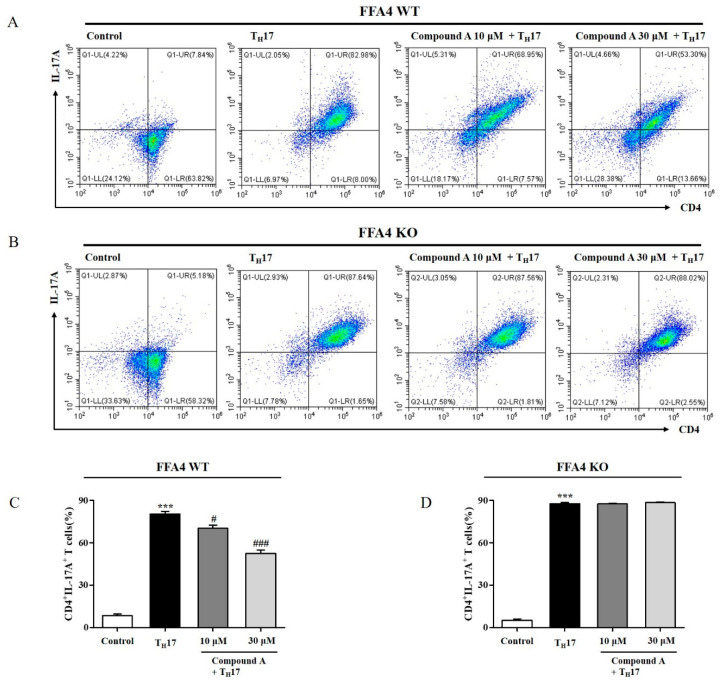
Administration of Compound A suppressed differentiation of naïve T cells into T_H_17 cells in FFA4 WT mice, but not in KO mice. CD4^+^ T cells isolated from splenocytes of FFA4 WT (**A**,**C**) or FFA4 KO mice (**B**,**D**) were incubated in T_H_17 differentiation media on plates coated with anti-mouse CD3 for 5 days. (**A**,**B**) Flow cytometry results. (**C**,**D**) Histogram of the CD4^+^IL-17A^+^ cell population. Data were tested for Gaussian distribution using the KS normality test. The results are presented as the mean ± SEM (n = 5). *** *p* < 0.001 vs. the vehicle-treated group, ^###^ *p* < 0.001, ^#^ *p* < 0.05 vs. the T_H_17 differentiation media-treated group.

Inflammatory T_H_17 cytokines play major roles in psoriasis pathogenesis. In addition, Compound A decreased the production of T_H_17 cytokines in the skin and lymph nodes. Therefore, we tested whether Compound A affected the differentiation of naïve T cells into T_H_17 cells by using CD4^+^ T cells from the spleen. T_H_17 differentiation was confirmed by an increase in the CD4^+^IL-17A^+^ T cell population; Compound A treatment decreased T_H_17 differentiation of T cells from FFA4 WT mice (Figure 7A,C). In T cells from FFA4 KO mice, T_H_17 differentiation was also induced, as shown by the increase in the CD4^+^IL-17A^+^ T cell population; however, Compound A treatment did not suppress T_H_17 differentiation of T cells from FFA4 KO mice (Figure 7B,D). Percentages of CD4^+^IL17^+^ T cells without T_H_17 induction from FFA4 KO mice were smaller than those from FFA4 WT mice (WT 7.8% vs. KO 5.2%), but T_H_17 differentiation media induced a higher percentage of CD4^+^IL17^+^ T cells in FFA4 KO mice than in FFA4 WT mice (WT 82% vs. KO 88%).

## 3. Discussion

N-3 PUFA supplementation has been studied in psoriasis as an adjuvant therapy, but many trials have shown unconvincing results [29]. In addition, n-3 PUFA supplementation can lead to side effects, such as gastrointestinal discomfort in elderly patients [30]. Therefore, it is important to determine the therapeutic target of n-3 PUFA to develop a highly specific regimen for psoriasis therapy without side effects. In this study, we identified FFA4 as a therapeutic target for psoriasis therapy using a selective FFA4 agonist, Compound A, in FFA4 KO mice [28]. Treatment with compound A ameliorated psoriasis skin lesions and suppressed the number of CD4^+^IL-17A^+^ T cells in the lymph nodes and the spleen in an FFA4-dependent manner. Furthermore, Compound A suppressed the differentiation of naïve T cells into T_H_17 cells via FFA4. Therefore, it is assumed that the activation of FFA4 by Compound A inhibits the action of pro-inflammatory effector T cells (T_H_1 and T_H_17) by suppressing the differentiation of CD4^+^IL-17A^+^ T cells. In contrast, Wannick et al. reported that oral administration of 50 mg/kg Compound A was not effective in alleviating imiquimod-induced psoriasis-like dermatitis; the dosage of Compound A used in the study was even higher than the dosage used in our study (30 mg/kg) [31]. Two factors differed between this study and previous studies. These include the mouse strains (C57BL/6 and BALB/c) and the administration route used (oral or intraperitoneal). However, Compound A was effective against insulin resistance and adipose tissue inflammation in C57BL/6 mice when administered orally [27], not taking into account these two factors. Although further investigation is necessary, previous studies by our group and others strongly support the health-promoting effects of n-3 PUFA through FFA4 in several inflammatory disease models [28,32,33,34,35,36].

Smaller weights of lymph nodes and spleens were observed in FFA4 KO mice compared to FFA4 WT mice (Figure 4 and Figure 6). In the spleen, percentages of CD4^+^IL17^+^ T cells in FFA4 KO mice were smaller than those in FFA4 WT mice (Figure 6 and Figure 7). These phenomena may occur through a biological adaptation to FFA4 gene deficiency. On the other hand, imiquimod-induced weight increases of lymph nodes were higher in FFA4 KO mice compared to FFA4 WT mice, and imiquimod-induced increases of CD4^+^IL17^+^ T cells in lymph nodes and spleens were much higher in FFA4 KO mice compared to FFA4 WT mice. This may occur due to the absence of suppressive effects of endogenous FFA4 activation. Multiple mechanisms have been suggested for the anti-inflammatory effects of n-3 PUFA [37]. The first mechanism involves the conversion of n-3 PUFA to pro-resolving lipid mediators (resolvins and protectins) [38,39]. Endogenous n-3 PUFA in a fat-1 transgenic mouse model protected against imiquimod-induced psoriasis-like inflammation via the IL-17/IL-23 axis [25]. Quin et al. showed that n-3 PUFA acted on T_H_17 cells to produce lower levels of inflammatory factors, including IL-17, IL-22 and IL-23, and stimulated Treg cells to produce higher anti-inflammatory factors, such as Foxp3 [25]. In fact, the n-3 PUFA-derived metabolite, resolvin E1, suppressed inflammatory cell infiltration and epidermal hyperplasia in psoriatic skin in an imiquimod-induced psoriasis mouse model [40]. The anti-psoriatic effects of resolvin E1 are mediated through the inhibition of leukotriene B_4_ and its receptor, BLT_1_, on neutrophils, which are increased in psoriatic skin lesions [40]. In addition, resolvin D5, a pro-resolving mediator, is upregulated in psoriatic lesions and shows inhibitory effects on the expression of IL-24 and S100A12 in keratinocytes [41]. Therefore, conversion of n-3 PUFA to specialized pro-resolving mediators may contribute to the beneficial effects of n-3 PUFA in psoriasis therapy.

The second mechanism involves n-3 PUFA competition for cyclooxygenases and lipoxygenases [37]. A substantial increase in n-3 PUFA-derived 5-lipoxygenase metabolites (leukotriene B_5_) and a significant decrease in leukotriene B_4_ production have been observed [42,43], thereby suggesting n-3 PUFA competition for lipoxygenase.

In this study, we found that FFA4 activation could be another target of n-3 PUFA in psoriasis therapy. Therefore, the beneficial effects of n-3 PUFA may partially result from the direct activation of FFA4, the indirect activation of the GPCRs of specialized pro-resolving mediators and competition with lipoxygenase substrates [37]. Due to the fact that psoriasis in humans is heterogeneous and complex, the use of a mouse model in the present study necessitates careful interpretation [3]. In the future, we hope that the application of FFA4 agonists in psoriasis could be a therapeutic option in clinical settings.

## 4. Materials and Methods

### 4.1. Materials

Compound A (3-[2-chloro-5-(trifluoromethoxy)phenyl]-3-azaspiro[5.5]undecane-9-acetic acid) was purchased from Cayman Chemical (Ann Arbor, MI, USA). Aldara cream, which contained imiquimod, was purchased from Dong-A ST (Seoul, Korea).

### 4.2. Animals

FFA4 knockout mice (TF0224) were purchased from Lexicon Pharmaceuticals (Woodlands, TX, USA) and backcrossed to BALB/c mice for 8 generations [25,26]. All animals were housed in a laboratory animal facility at Kyung Hee University and provided with food and water ad libitum. All animal procedures were conducted in compliance with the Kyung Hee University guidelines for animal care and use. The experimental protocol was evaluated and approved by the Institutional Animal Care and Use Committee of Kyung Hee University, with respect to the ethics of the procedures and care provided (KHSASP-21-213).

### 4.3. Induction of Psoriasis in BALB/c Mice and Compound A Administration

In this study, 8-week-old, BALB/c, male FFA4 WT and KO mice were randomly divided into six groups (n = 5) as follows: vehicle (Vaseline)-treated control FFA4 WT, imiquimod-treated FFA4 WT, Compound A/imiquimod-treated FFA4 WT, vehicle-treated control FFA4 KO, imiquimod-treated FFA4 KO and Compound A/imiquimod-treated FFA4 KO group. On Day 0, the skin on the back of the mice was shaved. Starting on Day 1, 62.5 mg of 5% imiquimod cream (Aldara cream, Dong-A ST) was applied daily for 6 days to induce psoriasis-like skin inflammation [44]. Compound A/imiquimod-treated FFA4 WT and KO group were administered an intraperitoneal injection of Compound A (30 mg/kg) 30 min before applying the imiquimod cream. The mice were sacrificed on Day 7 [45].

Inflammation of the back skin was scored daily based on the PASI during the experiment. The PASI scoring system includes erythema, scaling and thickening, each on a scale from 0 to 4 based on severity (0, none; 1, slight; 2, moderate; 3, marked; 4, very marked). The total score was obtained by accumulating the three index scores (score 0–12).

### 4.4. Histology and Immunohistochemistry

Specimens from the back skin were fixed in 10% formalin and dehydrated in a 30% sucrose solution for 24 h at 4 °C. The tissue samples were then embedded in O.C.T. compound. Sections (8 μm) were stained with hematoxylin and eosin (H&E). For immunohistochemical staining of Ki-67, the sections were washed with PBS containing 0.5% Tween-20 (PBS-T). The sections were blocked with PBS-T containing 5% bovine serum albumin for 30 min at room temperature. After blocking, the sections were labeled with Ki-67 recombinant rabbit monoclonal antibody (MA5-14520, Invitrogen, Carlsbad, CA, USA) for 1 h at room temperature. The VECTASTAIN^®^ Elite ABC Kit (PK-6101, Vector Laboratories, Burlingame, CA, USA) was used for staining. The peroxidase reaction product was visualized by incubating the slides with ImmPACT^®^ DAB Substrate (SK-4105, Vector Laboratories) for 10 min at room temperature. The slides were counterstained with hematoxylin.

### 4.5. T_H_17 Differentiation

Naïve CD4^+^ T cells were isolated from mouse splenocytes using magnetic beads (Naïve CD4^+^ T Cell Isolation Kit, Miltenyi Biotec, Bergisch Gladbach, Germany) [28]. They were induced to differentiate into T_H_17 cells by using a mouse T_H_17 cell differentiation kit (cat. CDK017, R&D systems, Minneapolis, MN, USA). Naïve CD4^+^ T cells were cultured with plate-coated anti-mouse CD3 in 12-well plates with T_H_17 differentiation media in X-VIVO 15 (Lonza, Basel, Switzerland) for 3 days. On Day 3, fresh T_H_17 differentiation media was added; the cells were then cultured for additional 2 days. On Day 5, the cells were collected and analyzed for T_H_17 differentiation via flow cytometry. Compound A (10 and 30 µM) was added to T_H_17 differentiation media to verify the effect.

### 4.6. Flow Cytometry

To determine T_H_17 cell population, single cells isolated from lymph nodes and spleens were stained with an FITC-labeled rat antibody against CD4 (cat. 11-0041-82, eBioscience, San Diego, CA, USA) at 4 °C for 15 min. The cells were then fixed at room temperature for 1 h using an IC fixation buffer (cat. 00-8222-49, eBioscience, San Diego, CA, USA). After fixation, the cells were permeabilized with permeabilization buffer (cat. 88-8824-00, eBioscience, San Diego, CA, USA) and stained at room temperature for 1 h with eFluor 660-labeled rat anti-IL-17A (cat. 50-7177-82, eBioscience, San Diego, CA, USA). The cells were analyzed using a CytoFLEX Flow cytometer (Beckman Coulter, Brea, CA, USA).

### 4.7. Quantitative Real-Time PCR

To assess the expression of inflammatory markers in the lymph nodes and skin of mice by RT-PCR, first-strand cDNA was synthesized from total RNA and isolated using a TRIzol reagent (Invitrogen, Waltham, MA, USA); total RNA was isolated from the lymph nodes and skin tissues and mRNA was transcribed to cDNA with Moloney Murine Leukemia Virus Reverse Transcriptase (Promega, Madison, WI, USA).

Quantitative PCR was performed using Thunderbird Next SYBR qPCR Mix (Toyobo, Osaka, Japan) and CFX Connect Real-Time System (Bio-Rad, Hercules, CA, USA). Specific primers for IL-17A (sense 5′-AGC TGG ACC ACC ACA TGA AT-3′, antisense 5′- AGC ATC TTC TCG ACC CTG AA-3′), IL-22 (sense 5′-CCG AGG AGT CAG TGC TAA GG-3′, antisense 5′-CAT GTA GGG CTG GAA CCT GT-3′), IL-23 (sense 5′-GAC CCA CAA GGA CTC AAG GA-3′, antisense 5′-CAT GGG GCT ATC AGG GAG TA-3′), IL-1β (sense 5′-CAG GCA GGC AGT ATC ACT CA-3′, antisense 5′-TGT CCT CAT CCT GGA AGG TC -3′), IFN-γ (sense 5′-CAC GGC ACA GTC ATT GAA AG-3′, antisense 5′-GTC ACC ATC CTT TTG CCA GT-3′), TNF-α (sense 5′-ACG GCA TGG ATC TCA AAG AC-3′, antisense 5′-AGA TAG CAA ATC GGC TGA CG-3′) and GAPDH (sense 5′-AAC TTT GGC ATT GTG GAA GG-3′, antisense 5′-GGA TGC AGG GAT GAT GTT CT-3′) were used to amplify gene fragments. Thermal-cycling conditions were as follows: one cycle at 95 °C for 4 min, 40 cycles at 95 °C for 30 s and at 57 °C for 30 s and one cycle at 95 °C for 30 s. The expression of individual genes was normalized to GAPDH levels.

### 4.8. Normality and Statistical Analysis

The results were expressed as means ± standard error of the mean (SEM) of five measurements for the animal experiments. The Kolmogorov–Smirnov (KS) test was performed to investigate whether the data passed the normality test. The statistical significance of the differences was determined by analysis of variance (ANOVA) and Tukey’s multiple comparison test. PASI scores were analyzed by two-way analysis of variance with Bonferroni post hoc test. Statistical significance was set at *p* values < 0.05. * indicates a significant difference compared to the vehicle-treated group; ^#^ indicates a significant difference compared to the imiquimod-treated group. Normality and analyses were performed using GraphPad Prism software (GraphPad Software, Inc., La Jolla, CA, USA).

## Data Availability

The study does not report any data.
